# Amylase/trypsin-inhibitor content and inhibitory activity of German common wheat landraces and modern varieties do not differ

**DOI:** 10.1038/s41538-025-00385-z

**Published:** 2025-02-20

**Authors:** Nora Jahn, Sabrina Geisslitz, Ulla Konradl, Klaus Fleissner, Katharina A. Scherf

**Affiliations:** 1 Department of Bioactive and Functional Food Chemistry, Institute of Applied Biosciences, Karlsruhe Institute of Technology (KIT), Adenauerring 20 a, 76131 Karlsruhe, Germany; 2 Leibniz Institute for Food Systems Biology at the Technical University of Munich, Lise-Meitner-Str. 34, 85354 Freising, Germany; 3 Bavarian State Research Center for Agriculture (LfL), Kleeberg 14, 94099 Ruhstorf an der Rott, Germany; 4 TUM School of Life Sciences, Professorship for Food Biopolymer Systems, Technical University of Munich, Lise-Meitner-Str. 34, 85354, Freising, Germany

**Keywords:** Proteomics, Agriculture

## Abstract

Amylase/trypsin-inhibitors (ATIs) are triggers for wheat-related disorders like baker’s asthma and non-celiac wheat sensitivity. With the rise of wheat-related disorders among the population, the hypothesis that breeding may have resulted in changes in the protein composition of wheat was put forward. The ATI content of 14 German common wheat landraces and six modern varieties harvested in three consecutive years was analyzed by liquid chromatography–tandem mass spectrometry, and the inhibitory activity against α-amylase was measured with an enzymatic assay. The mean ATI content and proportion of crude protein of both groups did not differ. There were also only small differences in the content and proportion of single ATIs. The mean values for the inhibitory activity of both groups were also similar. These results indicate that breeding might not have led to changes in the protein composition and landraces are unlikely to be better tolerated than modern varieties.

## Introduction

Wheat proteins can be classified into structural proteins, metabolic proteins, and storage proteins. The largest share of wheat proteins are the storage proteins (70–80%), also called gluten proteins. The metabolic proteins include the so-called amylase/trypsin-inhibitors (ATIs)^1,2^, which typically make up 2–6% of the total protein content of wheat^2^. They also occur in other gluten-containing cereals like rye and barley^3^. Several isoforms of ATIs have been identified and classified into four groups. The different names of the ATIs are based on their electrophoretic mobility and their solubility in chloroform/methanol (CM) mixtures. The first three groups mainly inhibit α-amylase from different species, excluding those of wheat. The monomeric inhibitor ATI 0.28 belongs to the first group. The second group of homodimeric inhibitors includes ATI 0.19 and ATI 0.53. The heterotetrameric inhibitors constitute the third group, which comprises the CM proteins CM1, CM2, CM3, CM16, and CM17. The fourth group includes CMX proteins which inhibit mainly trypsin^4^. The wheat amylase subtilisin inhibitor (WASI), wheat trypsin inhibitor (WTI), and the wheat chymotrypsin inhibitor (WCI) do not belong to any group but also occur in some species^2,5^.

Almost all ATIs have sections of homologous amino acid sequences. In addition, most ATIs have ten cysteine residues, which form five disulfide bridges. This conserved pattern of intrachain disulfide bonds leads to high stability in digestion and denaturation^4^. The ability of ATIs to inhibit digestive enzymes may lead to intestinal problems caused by insufficiently digested food components. They can also activate the toll-like receptor (TLR) 4 of the innate immune system^6,7^. These mechanisms are the reason why ATIs are triggers of several wheat-related disorders like baker’s asthma and non-celiac wheat sensitivity^4^. The relevance of ATIs in the context of various diseases requires quantitative analysis methods.

ATIs can be extracted as part of the Osborne fractionation from flour with aqueous buffers^8^ along with other enzymes and enzyme-inhibitors in the albumin and globulin (ALGL) fraction. Geisslitz et al.^2^ developed a targeted liquid chromatography–tandem mass spectrometry (LC–MS/MS) method based on stable isotope dilution analysis to quantitate 13 ATIs in ancient and modern *Triticum* species. For the inhibitory activity, there are several enzymatic assays available to measure the activity against α-amylase^9–11^ and trypsin^11,12^. Bioactivity is measured using an assay based on the release of chemokines and cytokines through activation of TLR4^7^.

There are possibilities to reduce the ATI content in the grain. Technologies like RNA-interference and CRISPR-Cas9 can be used to disrupt ATI expression^13^. Breeding can also be used to obtain varieties with low amounts of ATIs^4,14–16^. This brings up the question if the breeding process has already led to changes in the protein composition, especially in the ATI content, potentially increasing the immunoreactive potential of modern varieties^17^. This hypothesis has recently contributed to the rediscovery of old grain varieties like wheat landraces. Although the yield is usually lower compared to modern varieties, landraces are able to adapt better to challenging environments and shifting climate conditions. They are, therefore, suitable to be grown in local regions in a sustainable way. Wheat landraces are often associated with good flavor and taste^18^. Some consumers also report better tolerability of products made out of landraces^19^.

There are only a few studies about the ATI content and activity in old common wheat varieties. Call et al.^20^ evaluated the effects of breeding on a sample set of common wheat originating from 1850 to 2016. No trends were observed in ATI content and inhibitory activity against trypsin. Geisslitz et al.^14^ quantitated 13 different ATIs in 60 German hexaploid winter wheat varieties of old (first registered from 1891 to 1950) and modern (1851–2010) varieties. No difference in ATI content and distribution was found.

Although these studies included a large diversity of different varieties of various wheat species, none of them investigated common wheat landraces. At present, there is no study that has examined such a wide variety of 14 different wheat landraces, except our earlier study about their protein composition and baking volume^21^. The inhibitory activity towards α-amylase and the content and distribution of ATIs in common wheat landraces have not yet been investigated. This is why we determined the ATI content and inhibitory activity of 14 German common wheat landraces and six modern varieties grown under organic conditions for three consecutive years in this study. The aim of this study was to find out if the ATI content, composition, and inhibitory activity of landraces differ compared to modern varieties and if landraces might, therefore, be beneficial for people with wheat-related disorders.

## Results and discussion

### Total ATI content of wheat landraces and modern varieties

**Fig. 1:**
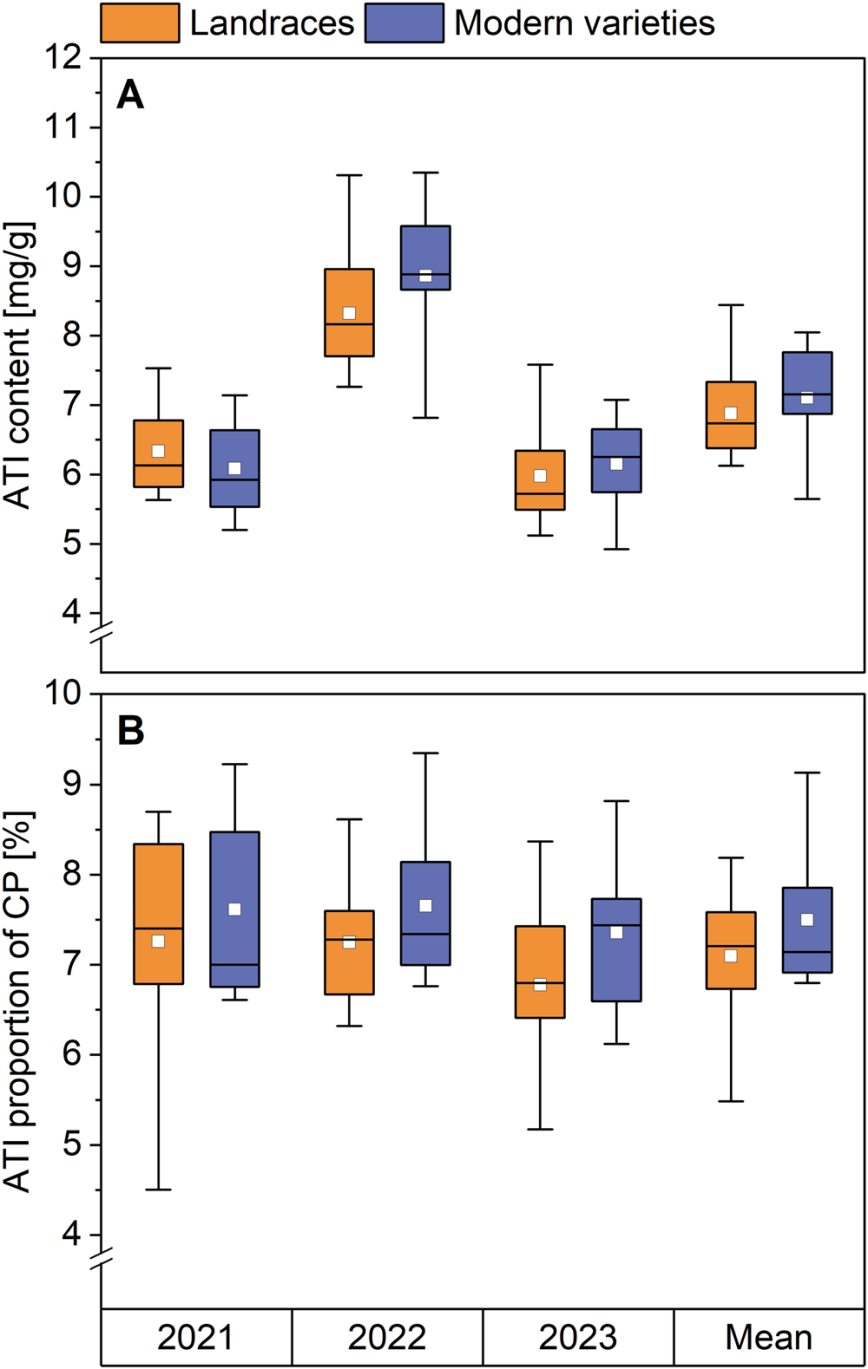
Amylase/trypsin-inhibitor (ATI) content (A) and proportion of crude protein (B) of landraces (*n* = 14) and modern varieties (*n* = 5–6). Boxplots display the interquartile range (box), mean value (white square), median (horizontal line), and minima and maxima (whiskers). CP crude protein.

The total ATI content of all samples (Supplementary Table 1) was calculated as the sum of all 13 ATIs (Fig. 1A, Supplementary Table 2A). In 2021 and 2023, the total ATI content of landraces and modern varieties was similar. For 2021, the landraces had an ATI content between 5.7 and 7.5 mg/g, while modern varieties had between 5.2 and 7.1 mg/g. For 2023, the content was 5.1–6.8 and 4.9–7.1 mg/g, respectively. In 2022, the ATI content reached the highest values. Landraces had between 7.3 and 10.3 mg/g of ATIs and modern varieties between 6.8 and 10.4 mg/g. To allow a better comparison, the ATI values are also presented as a proportion of crude protein (Fig. 1B; Supplementary Table 2B). The crude protein content of all samples is already reported by Jahn et al. ^21^ (Supplementary Table 3). In contrast to the absolute content, the ATI proportions were more similar to each other in the individual years. This result is due to the fact that both ATI and crude protein content were at their highest in 2022. The correlation coefficient of *r* = 0.764 showed a medium correlation between the ATI and the crude protein content. The mean ATI proportions for the years 2021, 2022, and 2023 were 7.3%, 7.2%, and 6.8% for landraces and 7.6%, 7.7%, and 7.4% for modern varieties, respectively. This resulted in mean values of 7.1% and 7.5% for all 3 years. In general, our ATI contents and proportions were in similar ranges compared to other studies. Common wheat samples reported by Geisslitz et al.^2^ had values of 3.5–4.5 mg/g and 3.3–5.3%. The 180 samples analyzed by Geisslitz et al.^14^ had ATI contents of 3.8–5.5 mg/g which led to proportions of 2.4–8.2%.

All in all, no significant differences (one-way analysis of variance (ANOVA), Tukey’s Test *p* < 0.05) were found in the absolute ATI content nor in the proportion of landraces and modern varieties in the individual years and in the mean of all years. Geisslitz et al.^14^, who analyzed 60 common (winter) wheat varieties originating from 1891 to 2010 from three harvest years also found no changes in ATI content from 1891 to 1970 and then a slightly decreasing trend. The ATI proportion of crude protein showed no changes from old to modern varieties as well. Call et al.^20^ also found no significant trend for the ATI content of common wheat varieties originating from 1850 to 2016.

The landrace EGH had the highest absolute mean value of ATIs with 8.4 mg/g (and also the highest value in each year separately: 7.4 mg/g in 2021, 10.3 mg/g in 2022 and 7.6 g/g in 2023). The modern variety RGT had the highest proportions of ATIs in all 3 years with 9.2% in 2021, 9.3% in 2022 and 8.8% in 2023, also resulting in the highest mean value (8.4%) over all 3 years.

The modern variety ELX has the lowest ATI content in all 3 years (5.2, 6.8, and 4.9 mg/g) and therefore also the lowest mean value (5.6 mg/g). The landrace WEI had the lowest proportions of ATI in the years 2021 and 2023 (4.5% and 5.2%) and one of the five lowest values in 2022 (6.8%), resulting in the lowest mean value (5.0%). Geisslitz et al.^14^ also identified a modern variety (Dekan; originating from 2001 to 2010) with a low ATI content in all 3 harvest years.

ATIs belong to the water and salt-soluble ALGL. The ATI proportion based on the ALGL content (reported in Jahn et al.^21^, Supplementary Table 4) in all three years was about one-third for landraces (36.6%, 38.3%, and 32.6%) and for modern varieties (30.9%, 38.3%, and 36.1%) (Supplementary Table 2C). The mean values of both groups did not differ (35.6% and 35.4%, respectively). This is in accordance with Geisslitz et al.^14^ who also did not find significant differences between the ATI proportions based on the ALGL content of old and modern varieties. We found a strong correlation between the ATI and the ALGL content (*r* = 0.827). Since there was no difference in ATI content and proportions between landraces and modern varieties, the ATI composition was examined more closely.

### ATI distribution

**Fig. 2:**
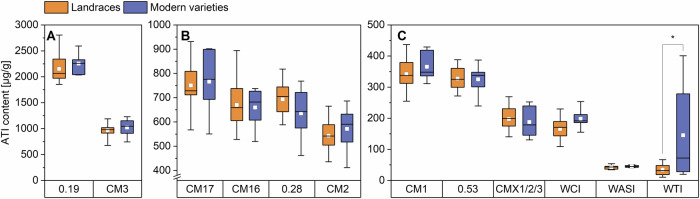
Amylase/trypsin-inhibitor (ATI) content of single ATIs of landraces (*n* = 14) and modern varieties (*n* = 6). ATIs 0.19 and CM3 (**A**), ATIs CM17, CM16, 0.28 and CM2 (**B**), ATIs CM1, 0.53, CMX1/2/3, WCI, WASI and WTI (**C**). The results are displayed as in Fig. 1. Asterisks indicate significant differences between landraces and modern varieties (one-way ANOVA, Tukey’s test at *p* < 0.05). CM chloroform/methanol, WASI wheat amylase subtilisin inhibitor, WCI wheat chymotrypsin inhibitor, and WTI wheat trypsin inhibitor.

**Fig. 3:**
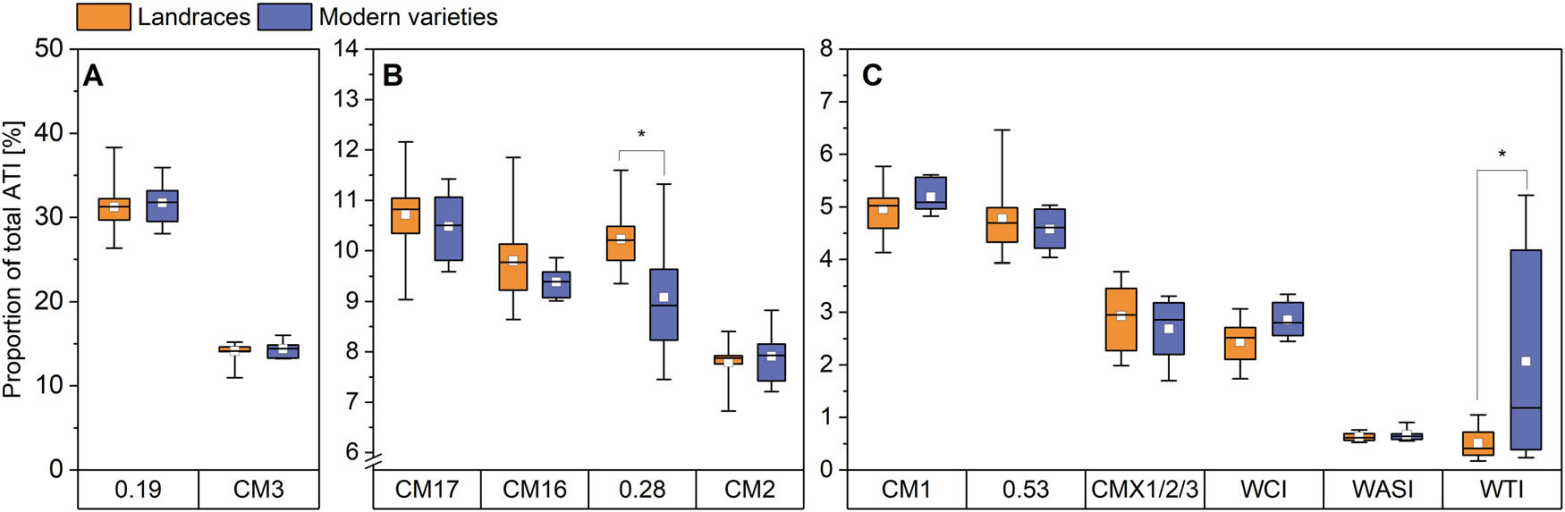
Amylase/trypsin-inhibitor (ATI) proportion of single ATIs of landraces (*n* = 14) and modern varieties (*n* = 6). ATIs 0.19 and CM3 (**A**), ATIs CM17, CM16, 0.28 and CM2 (**B**), ATIs CM1, 0.53, CMX1/2/3, WCI, WASI and WTI (**C**). The results are displayed as in Fig. 1. Asterisks indicate significant differences between landraces and modern varieties (one-way ANOVA, Tukey’s test at *p* < 0.05). CM chloroform/methanol, WASI wheat amylase subtilisin inhibitor, WCI wheat chymotrypsin inhibitor, and WTI wheat trypsin inhibitor.

The mean ATI contents and the distribution of the single ATIs of landraces and modern varieties are displayed in Figs. 2 and 3. The values for all samples of all harvest years are summarized in Supplementary Tables 5–16.

With mean values of 2152 µg/g for the landraces and 2253 µg/g for the modern varieties, the absolute content of 0.19 did not differ between the groups. The content of 0.19 was more than two times higher than those of Geisslitz et al.^2,14^ (813–1126g and 679–1304 µg/g). The higher values probably result from the different peptides that were used for quantitation. Our quantifier (P/IS22) is unique for 0.19 in contrast to the one used in Geisslitz et al.^2,14^, which is present in both 0.19 and 0.53. Nevertheless, Geisslitz et al.^14^ also found no difference in the content of 0.19 in old and modern varieties. With 31%, the 0.19 makes up the largest share of the total ATI content, which is also in line with the previous studies^2,14^ and the study of Sielaff et al.^15^, who analyzed several ATIs with a method that involves artificial proteins that release the target peptides during tryptic hydrolysis.

With 969 and 1021 µg/g, respectively, for landraces and modern varieties, there was also no difference between the two groups for CM3. The mean content of CM3 in other studies was between 665 and 911 µg/g^2^ and between 678 and 1094 µg/g^14^ and, therefore, in the same range. The absolute content of CM3 in the study of Geisslitz et al.^14^ was slightly lower in modern varieties compared to varieties from older decades. For both landraces and modern varieties, 0.19 and CM3 together made up around 45% of the total ATI content. With 40%, Geisslitz et al.^2^ found a similar distribution to the one in our study.

In our study, the mean value for CM17 was 751 µg/g for the landraces and 766 µg/g for the modern varieties. The mean content of CM16 was 670 µg/g for the landraces and 651 µg/g for the modern varieties, indicating similar values for CM16 and CM17, which is in accordance with other studies^2,14,15^. For both ATIs, the absolute contents were in the same range as reported before (CM17: 415–724 and 415–724 µg/g; CM16: 347–422 and 354–645 µg/g)^2,14^.

For 0.28, the mean content was 694 and 635 µg/g and for CM2 it was 544 and 572 µg/g, respectively, indicating slightly higher values compared to earlier studies (0.28: 307–411 and 314–561 µg/g; CM2: 302–407 and 281–541 µg/g)^2,14^. No difference between landraces and modern varieties could be observed, which is again in line with Geisslitz et al.^14^. Together, this equals a share of approx. 40% in sum for CM17, CM16, 0.28 and CM2 for both the landraces and the modern varieties, which is in accordance with the results of Geisslitz et al.^2^.

The absolute content of CM1 and 0.53 in our study was similar to each other and also similar in both groups. The content of CM1 was 342 µg/g and that of 0.53 was 328 µg/g in the group of landraces. For the modern varieties, this content was 365 and 325 µg/g, respectively. For CM1, Geisslitz et al.^14^ also found no differences between old and modern varieties, but for 0.53 they found a significant difference between varieties from 1901 to 1910 and those from 1991 to 2000. Although the absolute contents of these ATIs in our study were slightly higher compared to those from Geisslitz et al.^2^ (CM1: 171–226 and 160–271 µg/g; 0.53: 149–239 and 160–266 µg/g), CM1 and 0.53 each contribute about 5% to the total ATI content of landraces and modern varieties.

There were also no differences between landraces and modern varieties for the other ATIs (except WTI), either. These findings are consistent with those of Geisslitz et al.^14^. The mean content for CMX1/2/3 was 198 µg/g for the landraces and 188 µg/g for the modern varieties. For WCI, the landraces had a mean content of 166 µg/g and the modern varieties of 198 µg/g. Mean contents of CMX1/2/3 and WCI are in the same range as the ones from Geisslitz et al.^2,14^ (CMX1/2/3: 58–145 and 81–282 µg/g; WCI: 69–139 and 116–201 µg/g).

The mean content for landraces for WASI was 43 µg/g, and that of modern varieties was 45 µg/g and, therefore, on average, slightly lower than those of Geisslitz et al.^2,14^ (86–125 and 42–81 µg/g). Landraces had 35 µg/g and modern varieties 145 µg/g for WTI, showing a significant difference (one-way ANOVA with Tukey’s Test *p* < 0.05). The high value for the modern varieties was mainly due to two varieties (RGT and BOS), which had high contents in all three harvest years. A wide range of the WTI content of the modern varieties was also found in the two studies of Geisslitz et al. (15–282^2^ and 19–146 µg/g^14^). Without these two samples, the mean of the modern varieties would be 48 µg/g and in the same range as the one of the landraces. CMX1/2/3, WCI, WASI, and WTI had proportions between around 0.5% and 3% each and together made up 5–10% of total ATI content, which is also in accordance with Geisslitz et al.^2,14^.

All in all, the content and the distribution of the different ATIs relative to the total ATI content were very similar for all samples, regardless of whether it was a landrace or a modern variety. A few samples stood out because of higher or lower proportions compared to the others (Supplementary Fig. 1). The landrace WEI had the highest proportion of ATI 0.19 (38.3%) and the lowest proportion of CM3 (11.0%). The high mean content of WTI for the modern varieties (145 µg/g) was caused by the two varieties RGT (401 µg/g) and BOS (278 µg/g), resulting in a proportion of 5.2% and 4.2% of total ATI. In all 3 years, the values for the two were considerably higher than for the other modern varieties and landraces.

**Table 1:** LC-MS/MS parameters for the quantitation of amylase/trypsin-inhibitors (ATIs) by parallel reaction monitoring (PRM)

Peptide/ internal standard (P/IS)	ATI	Amino acid sequence^a^	Precursor *m/z*		Product ions	Collision energy (eV)
			P	IS		
P1/IS1	0.28	LQ**C**VGSQV*PEA*VLR	778.4167	784.4305	b3/y6/y7/y10/y11/y12	27
P2/IS2	0.28	LTAASVPEVC*K	587.8105	591.8176	y3/y5/y6/y7/y8/y9	24
P3/IS3	0.19 + 0.53	LQ**C**NGSQV*PEA*VLR LQ**C**NGSQVPEAVL*R	785.9039 785.9039	791.9177 790.9081	y6/y7/y8/y10/y11/y12 y6/y7/y8/y10/y11/y12	27 27
P4/IS4	0.19 + 0.53	LTAASITAVC*R	581.8161	586.8202	y6/y7/y8/y10/y11/y12	27
P5/IS5	0.53	EHGVSEGQAGTGAFPSC*R	616.2761^b^	619.6122^b^	b8/y4/y5/y6/y7/y9	27
P6/IS6	CM1	SDPNSSVL*K	473.7456	477.7527	y7^c^/y5/y6/y7	33
P7	CM2	EYVAQQT**C**GVGIVGSPVSTEPGNTPR	1352.1558	–	y6/y7/y9/y11/y12/y13	30
P8/IS8	CM2	TSDPNSGVL*K	509.2642	513.2713	b3/y7^c^/y8^c^/y7/y8/y9	18
P9/IS9	CM3	YFIALPVPSQPVDP*R	849.9643	854.9685	b3/b4/y8/y10/y11/y12	27
P10/IS10	CM3	SGNVGESGLIDL*PGC*PR	864.4227	870.4365	b3/y5/y6/y7/y11/y13	27
P11/IS11	CM16	DYVEQQAC*R	584.7562	589.7603	b2/y7^c^/y5/y6/y7	18
P12/IS12	CM16	QQ**CC**GELANIPQQC*R	931.4087	936.4129	y5/y6/y7/y8/y9/y11	27
P12c/IS12c	CM16	**Q**Q**CC**GELANIPQQC*R	922.8955	927.8996	b3/b6/y5/y7/y8/y9	27
P13/IS13	CM17	NYVEEQAC*R	584.7562	589.7603	b2/b3/y8^c^/y5/y6/y7	18
P14/IS14	WASI	HVITGPV*R	439.7640	444.7681	b3/y4/y5/y6/y7	22
P15/IS15	WASI	YSGAEVHEY*K	591.7749	595.7820	y8^c^/y9^c^/y4/y6/y8/y9	20
P16/IS16	CMX1/2/3	EFIAGIVG*R	481.2769	486.2810	b3/y3/y4/y5/y6/y7	22
P17/IS17	WCI	ELAAISSNC*R	560.7744	565.7785	b3/b4/y5/y6/y7/y8	20
P18/IS18	WCI	AFPPSQSQGGGPPQPPLAP*R	993.5132	998.5174	y6/y9/y10/y11/y12/y14	27
P19/IS19	WTI	ELEAVSEEC*R	611.2744	616.2786	b4/y4/y5/y6/y7/y8	18
P20/IS20	WTI	LEGVPEG**C**T*R	559.2690	564.2731	b2/b3/b4/y6^c^/y6/y8	20
P21/IS21	CMX1/3	GSLLQDMS*R	503.7529	508.7571	b3/y6^c^/y7^c^/y4/y5/y6	20
P22/IS22	0.19	EHGAQEGQAGTGAFP*R	538.2536^b^	541.5897^b^	b5/b8/y5/y6/y7/y8	27

*CM* chloroform/methanol, *WASI* wheat amylase subtilisin inhibitor, *WCI* wheat chymotrypsin inhibitor, *WTI* wheat trypsin inhibitor.

^a^**C**, S-carboxyamidomethylcysteine; **Q**, pyroglutamyl; *P, proline (^13^C_5_, ^15^N); *V, valine (^13^C_5_, ^15^N); *K, lysine (^13^C_6_, ^15^N_2_); *R, arginine (^13^C_6_, ^15^N_4_).

^b^Precursors were 3^+^, all other ones 2^+^.

^c^Product ions were 2^+^, all other ones 1^+^.

No significant differences in the content of individual ATIs (one-way ANOVA with Tukey’s Test *p* < 0.05) were observed between the two groups, except for WTI, where the modern varieties had a higher mean value due to the two samples that were mentioned above. The same was true for the proportions of individual ATIs relative to total ATI, with the two exceptions of 0.28, where the proportion was significantly higher in landraces and again the WTI which was significantly higher for the modern varieties. Geisslitz et al.^14^ also found no significant differences between old and modern varieties for eight of the twelve ATIs. For the other four ATIs CM3, CM17, CM16, and 0.53, the modern varieties tended to have a slightly lower content.

### Inhibitory activity

**Fig. 4:**
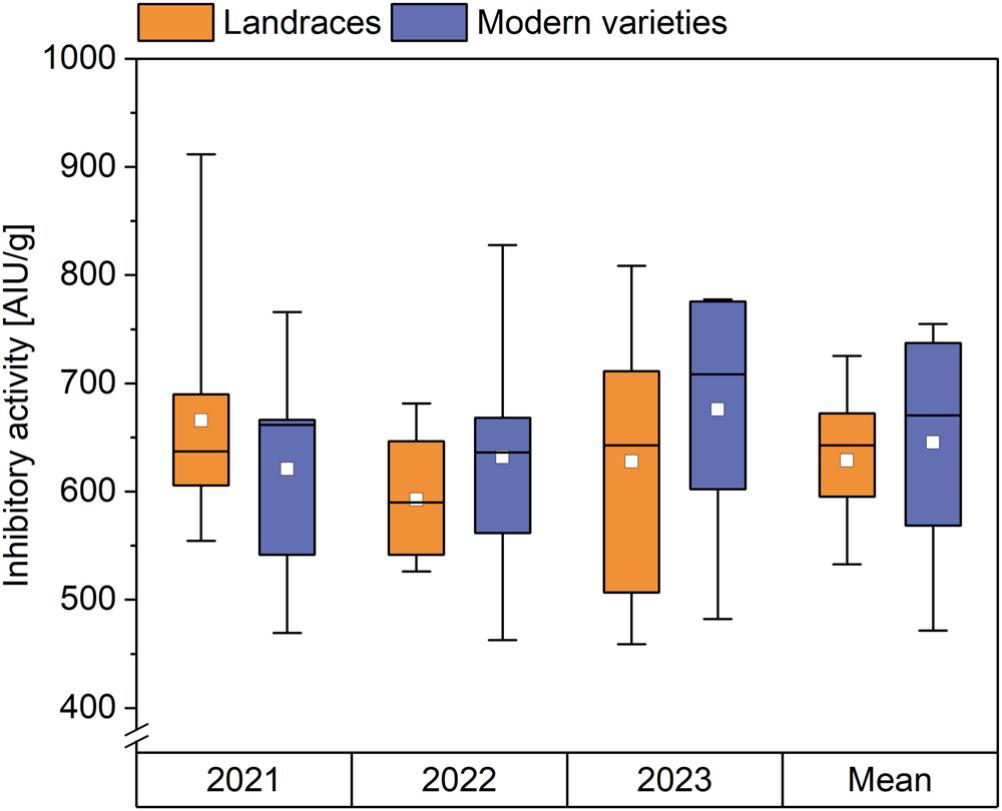
Inhibitory activity of landraces (*n* = 14) and modern varieties (*n* = 5–6) against porcine pancreas α-amylase. The results are displayed as in Fig. 1. AIU amylase inhibiting units.

The inhibitory activity towards porcine pancreas α-amylase of all samples was measured as an indication of activity towards human saliva amylase^9^. The inhibitory activity did not differ between landraces and modern varieties (Fig. 4, Supplementary Table 17). In 2021, the inhibitory activity of the landraces was 554–912 AIU/g, that of the modern varieties 469–766 AIU/g. In 2022, the activities of the landraces were 526–681 AIU/g, those of the modern varieties 463–828 AIU/g. In 2023, these values were 459–809 AIU/g for the landraces and 482–778 AIU/g for the modern varieties. The mean ATI inhibitory activity against porcine pancreas α-amylase of landraces and modern varieties over all 3 years was between 471 and 755 AIU/g. This range is smaller than the one of the eight common wheat samples from three locations analyzed in a previous study (580–2885)^9^.

We did not observe differences between the mean activity of landraces (629 AIU/g) and the one of modern varieties (646 AIU/g). Simonetti et al.^11^ analyzed the inhibitory activity of modern and ancient wheat genotypes with different ploidy levels including two heritage and two modern common wheat varieties. No trend was observed between the inhibitory activities of these varieties, which matches our findings. Further, we found no variety that stood out due to particularly high or low activity, except the modern variety ELX, which had low activities in all 3 years (469, 463, and 482 AIU/g).

### Effect of harvest year and variety

In 2022, the mean ATI content of landraces and modern varieties was the highest (8.3 and 8.9 mg/g) compared to 2021 and 2023. The content did not differ in 2021 (6.3 and 6.1 mg/g) and 2023 (6.0 and 6.3 mg/g). Geisslitz et al.^14^ also found differences between the three harvest years, having mean values of 6.3, 5.0, and 2.3 mg/g for the years 2015, 2017, and 2019. The environmental effect was confirmed by running a two-way ANOVA, which stated that the harvest year had a bigger influence on the ATI content (*F* = 52.7; *p* < 0.0001, Supplementary Table 18A) than the genetic background (modern variety or landrace; *F* = 0.45; *p* = 0.5038). For the inhibitory activity against α-amylase, there was no trend for the different years. There was no significant influence for both the harvest year (*F* = 0.74; *p* = 0.4821) and the variety (*F* = 0.25; *p* = 0.6206, two-way ANOVA, Supplementary Table 18B) on the inhibitory activity.

### ATI content and inhibitory activity in landraces and modern varieties

**Fig. 5:**
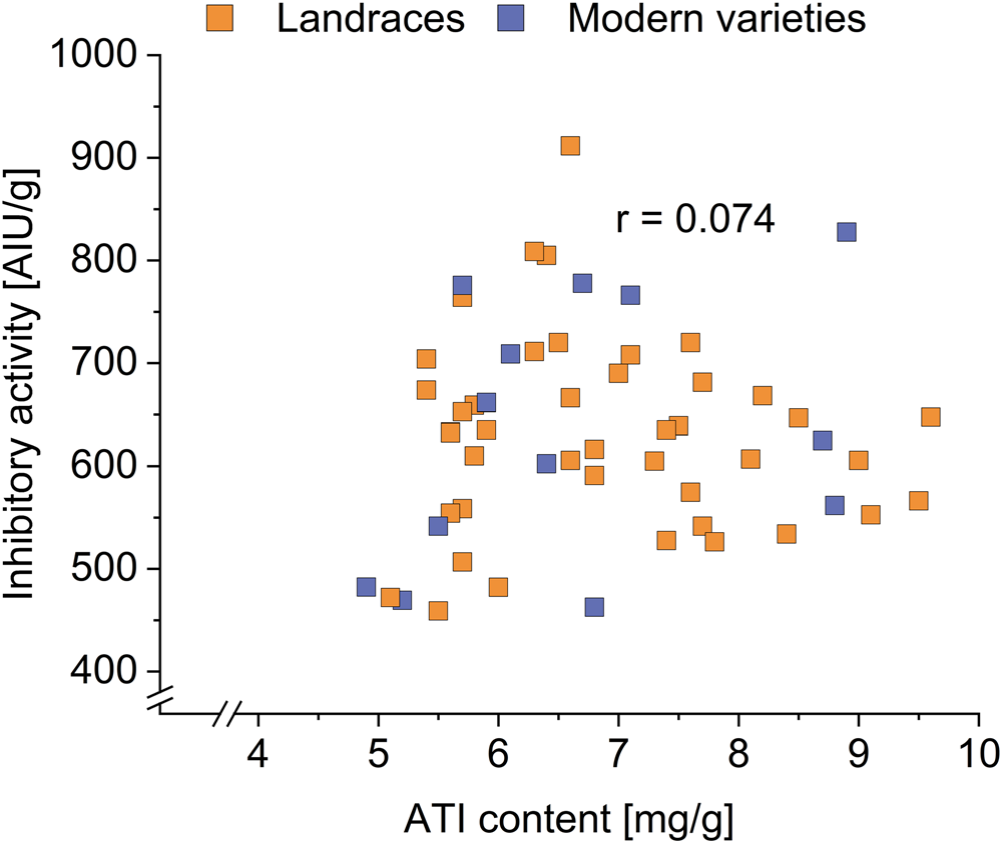
Correlation diagram of amylase/trypsin-inhibitor (ATI) content and inhibitory activity against porcine pancreas α-amylase. All varieties of all 3 years are displayed. AIU amylase inhibiting units.

The correlation plot between the ATI content and inhibitory activity of all varieties from all harvest years showed no cluster or linear relation (Fig. 5). The Pearson correlation coefficient indicated no correlation (*r* = 0.074; Supplementary Table 19A).

Pearson correlations were also performed with the mean values (Supplementary Fig. 2). A weak correlation was found (*r* = 0.555). Since ATI 0.19 and CM3 are known to be the most bioactive ones^6^ (and these two ATIs also have the highest content in our study), a correlation analysis was also carried out between the mean inhibitory activity and content of these two ATIs. No correlation was found for 0.19 (*r* = 0.229) and a weak correlation was found for CM3 (*r* = 0.629). Because the inhibitory activity against α-amylase was measured, a correlation analysis just with the mean content and activity of the ATIs that mainly inhibit α-amylase (all ATIs except WASI, WTI, WCI, and CMX1/2/3) was carried out. Again, no correlation (*r* = 0.503, Supplementary Table 19B) was found. These results are in accordance with Jahn et al.^9^, who also showed no correlation between the ATI content and the inhibitory activity (*r* = 0.23). This could mean that the inhibitory activity and the content of ATI are independent of each other and that not every ATI has the same activity. A correlation might be found when considering also the inhibitory activity against trypsin and the bioactivity, but these results highlight that many factors and parameters need to be considered when looking at the immunoreactive potential of proteins.

**Fig. 6:**
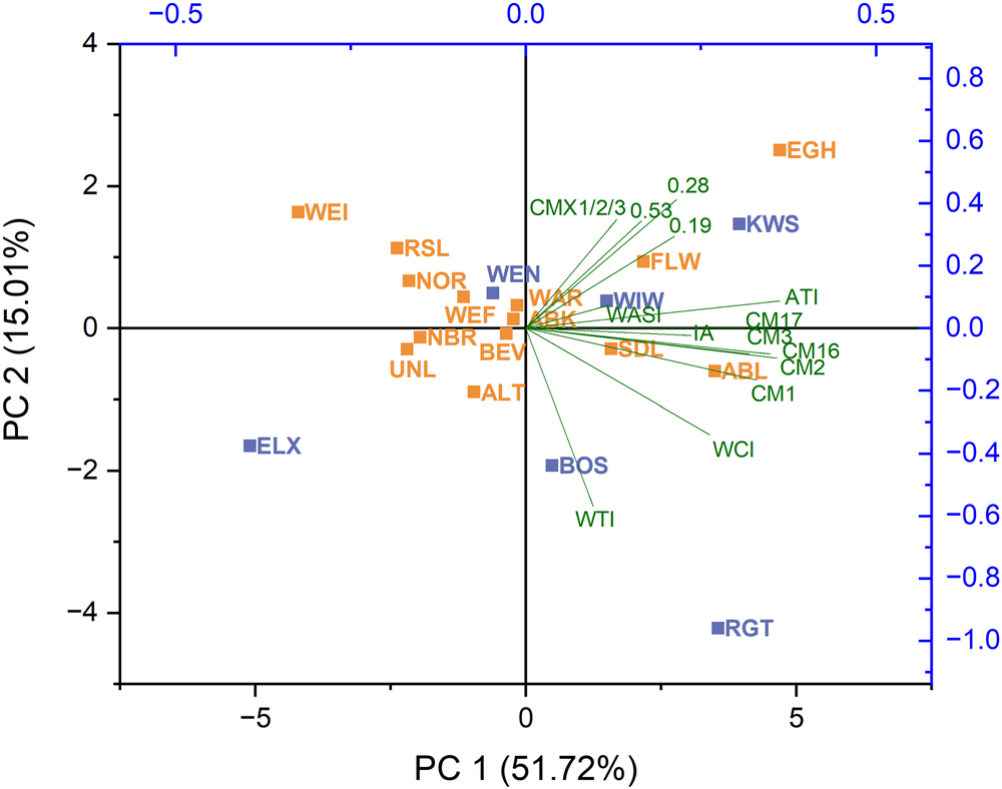
Principal component (PC) analysis biplot based on total amylase/trypsin-inhibitor (ATI) content, the content of all single ATIs, and the inhibitory activity (IA) against porcine pancreas α-amylase. Abbreviations for the varieties can be found in Supplementary Table 19. CM chloroform/methanol, WASI wheat amylase subtilisin inhibitor, WCI wheat chymotrypsin inhibitor, WTI wheat trypsin inhibitor.

A principal component analysis (PCA) was performed based on the inhibitory activity, the total ATI content and the content of individual ATIs (Fig. 6). PC1 and PC2 accounted for 67% of the total variance. The CM-types mostly contributed to PC1, while PC2 was mainly composed of WTI, CMX1/2/3, and the monomeric and dimeric ATIs. The score plot showed no cluster formation for the two groups of landraces and modern varieties. All loadings pointed in the same direction to the positive side of PC1, while ten out of 14 landraces were projected on the negative side. A few varieties stood out. The modern varieties BOS and RGT were both placed in the direction of the loading of WTI, visualizing that these two varieties had a relatively high content of WTI. The landrace WEI was displayed in the opposite direction because it had the lowest WTI content of all varieties. The landraces EGH, KWS and FLW had the highest content of ATI 0.19 and were therefore located close to its loading. EGH stood out the most, having the highest content of ATI 0.19 and also the highest content of 0.28, whose loading points are in the same direction. The modern variety ELX was located at the opposite end because it had the lowest content of ATI 0.28. According to the analyzed parameters it was not possible to distinguish between landraces and modern varieties.

Although no difference was found between landraces and modern varieties concerning the ATIs, there were some varieties that stood out. The variety with the lowest absolute mean ATI content was a modern variety (ELX), which also had the lowest content in all three years separately. Even though there was no correlation between inhibitory activity and ATI content in this study, this variety also had the lowest inhibitory activity. This variety is a promising candidate for breeding programs and might be further interesting for individuals with non-celiac wheat sensitivity. The landrace WEI had the lowest ATI proportion relative to crude protein.

All in all, we found similar total ATI contents, distribution of single ATIs, and inhibitory activities in landraces and modern varieties. One limitation of our study is that the statistical evaluation must be handled cautiously because we had 14 landraces and six modern varieties. The imbalance due to the different group sizes and the relatively small sample amount, in general, requires critical interpretation. However, other studies came to the same conclusions as we did. Call et al.^20^ found similar amounts of ATIs in old and modern common wheat varieties. El Hassouni et al.^16^ discovered no trends regarding the ATI content in varieties originating from numerous decades. Geisslitz et al.^14^ confirmed that old and modern varieties were comparable in terms of ATI content and composition. D’Amico et al.^22^ also concluded that landraces did not seem to be better tolerable based on their protein composition, since the content of ATIs was comparable to modern varieties. Taken together, these studies suggest that breeding did not lead to a significantly different ATI content and composition in modern varieties compared to old varieties (landraces).

## Methods

### Grain Samples

Fourteen German common wheat (*Triticum aestivum* L.) landraces and six modern varieties were cultivated under organic conditions (without fertilization) at the Bavarian State Research Center for Agriculture (Ruhstorf an der Rott, Germany) and harvested in 3 consecutive years (2021–2023), with the exception of one variety which was only available in 2 years (WIW) (Supplementary Table 1). The varieties were grown in a randomized complete block design in triplicate but harvested and milled together with a Quadrumat Junior mill (Brabender, Duisburg, Germany) to obtain type 550 flours (ash content of 0.51–0.63% based on dry matter) according to the German flour classification system.

### Reagents

All reagents were of analytical grade or higher and obtained from Carl Roth (Karlsruhe, Germany), VWR Chemicals (Radnor, PA, USA), and Thermo Fisher Scientific (Waltham, MA, USA). EnzChek^TM^ Ultra Amylase Assay was obtained from Thermo Fisher Scientific. Trypsin (TPCK-treated, bovine pancreas) and porcine pancreas amylase (A3176) were from Sigma-Aldrich (St. Louis, MO, USA). Peptides were synthesized by GenScript (Piscataway, NJ, USA) as unlabeled peptides (P1–P22) and as stable isotope labeled internal standards (IS1–IS22) with two heavy amino acids (IS1, 3, 10) or C-terminal heavy K or R (IS2–6, 8, 9, 11–22) (^13^C and ^15^N) (Table 1). For stock solutions, the peptides were solubilized in water (1 mg/mL) and stored at −80 °C prior to use.

### ATI content by LC–MS/MS

The method to quantitate ATIs in wheat described in Geisslitz et al.^2^ was adapted to a Q Exactive Plus Orbitrap (Thermo Fisher Scientific) mass spectrometry system coupled to a Vanquish ultra-high-performance liquid chromatography system (Thermo Fisher Scientific). The method was slightly modified as not all internal standards were included, but new ones were added, and some heavy-labeled amino acids varied (Table 1).

#### Sample preparation

Flour (50 mg) was extracted twice with ammonium bicarbonate solution (0.5 mL, 50 mmol/L, pH 7.8) for 30 min at 22 °C using a magnetic stirrer. After every extraction step, the suspensions were centrifuged (25 min, 22 °C, 3750 rcf) and the supernatants were combined. Using a rotational vacuum concentrator (Christ, Osterode, Germany), the extracts were then evaporated to dryness. The residue was dissolved in 350 µL of tris(hydroxymethyl)aminomethane (Tris)–HCl (0.5 mol/L, pH 8.5) and 350 µL of 1-propanol. Then 50 µL of standard solution, containing IS1–22, was added. The concentration of each IS in this solution was adjusted to the expected peptide content in the samples. After that, 50 µL of tris(2-carboxyethyl)phosphine (TCEP) was added (0.05 mol/L TCEP in 0.5 mol/L Tris–HCl, pH 8.5) and incubated for 30 min at 60 °C in a thermal shaker (Hettich Lab Technology, Tuttlingen, Germany) to perform the reduction of the disulfide bonds. Alkylation was performed in the same device by adding 100 µL of 2-chloracetamide (CAA) (0.5 mol/L CAA in 0.5 mol/L Tris–HCl, pH 8.5) and incubating for 45 min at 37 °C in the dark. The solvent was again removed by evaporation to dryness. Last, 0.5 mL of trypsin solution (enzyme-to-substrate ratio 1:30, 0.04 mol/L urea in 0.1 mol/L Tris–HCl, pH 7.8) was added for tryptic digestion for 18 h overnight at 37 °C in the dark. After that, the reaction was stopped with 5 µL of trifluoroacetic acid. The solution was removed by evaporation, and the residue was then dissolved in 1 mL of water containing 2% acetonitrile and 0.1% formic acid.

For calibration, two solutions (25–100 µg/mL of each peptide) were prepared from the stock solutions, solution 1 with P1–P22 and solution 2 with IS1–IS22. An aliquot of each solution 1 and 2 was reduced and alkylated similar to the samples and as described in Geisslitz et al.^2^. Alkylated solutions 1 and 2 (0.3–1.3 µg/mL of each peptide) were mixed in molar ratios *n*(P)/*n*(IS) between 9.1 and 0.1 (9 + 1, 7 + 1, 5 + 1, 3 + 1, 1 + 1, 1 + 3, 1 + 5, 1 + 7, and 1 + 9) and used for quantitation.

Limits of detection and quantitation were also determined according to Geisslitz et al.^2^ (Supplementary Table 20).

#### Absolute quantitation by parallel reaction monitoring (PRM)

The qualifier internal standard for CM2 (IS7) was not included anymore due to low stability in the solution, but P7 was kept to guarantee the correct identification of CM2. IS12 (CM16) had a different heavy label compared to the initial method and for IS3 (ATI 0.19 + 0.53), two peptides with varying labeling were used because the new internal standards (IS3 and IS12) were purchased with only one heavily labeled amino acid (heavy R) instead of two heavy amino acids. P21/IS21 was added as a specific peptide for CMX1/3 and P22/IS22 for 0.19. Skyline (version 23.1.0.380, MacCoss Lab Software, University of Washington, Seattle, WA, USA) was used to adapt the method^23^, and the same settings were applied as described previously^2^: Peptide settings: digestion: trypsin [KR I P]; max missed cleavages: 0; filter: min length: 8; max length: 26; exclude N-terminal AAs: 25; modifications: carbamidomethyl (C), oxidation (M), Gln → pyro-Glu (N-term Q). Transition settings: prediction: precursor mass: monoisotopic; product ion mass: monoisotopic; collision energy: Thermo; declustering potential: none; optimization library: none; compensation voltage: none; filter: precursor charges: 2, 3; ion charges: 1, 2; ion types: y, b; product ion selection: From ion 3 to last ion −1; instrument: min *m*/*z*: 150; max *m*/*z*: 1400; method match tolerance *m*/*z*: 0.6; full scan: MS1 filtering: none; MS/MS filtering: acquisition method: PRM; product mass analyzer: Orbitrap; resolving power: 17,500 at 400*m*/*z*.

An Aeris column (1.7 µm PEPTIDE XB-C18 10 nm, 150 × 2.1 mm) was used. The column oven temperature was 30 °C, and the flow rate was 0.2 mL/min. The solvents were A: water with 0.1% formic acid, B: acetonitrile with 0.1% formic acid. The gradient was 0–12 min 5–15% B, 12–18 min 15–30% B, 18–19 min 30–80% B, 19–21 min 80% B, 21–22 min 80–5% B, and 22–30 min 5% B. The ion source was operated in the positive electrospray ionization mode, and the following source parameters were set: sheath gas 35 a.u.; aux gas 10 a.u.; sweep gas 0 a.u.; spray voltage 3.00 kV; capillary temperature 350 °C and S-lens level 60. The MS2 parameters were set to: resolution: 17,500; automatic gain control target: 2e4; maximum injection time: 50 ms; isolation window: 1.6*m*/*z*. Collision energies were optimized by testing 18, 20, 22, 24, 27, and 30 (default setting in Skyline: 27) and the collision energy obtaining the highest peak intensity was used for the final method (Tab.1). The injection volume was 10 µL for the response curves and 2 µL for the samples.

#### Data analysis

Data analysis was performed using Skyline as described in Geisslitz et al.^2^. For the response lines, the peak area ratios were plotted against the molar ratios of the unlabeled peptide and labeled internal standard. For quantification, peak area ratios from three replicates with one injection each, were used. Selection of the peptides for quantitation is described in the next chapter.

#### Selection of peptides for ATI quantitation

For quantitation, one peptide (quantifier) was selected for each ATI (Table 1). If available, the other peptide(s) were used as a qualifier to support correct identification. The aim was to select two peptides per protein because of posttranslational modifications, but sometimes no other peptides were detected or had low intensities^2^.

For the ATIs CM1, CM2, and CM17, only one peptide was available, respectively, because no other unique peptides were detected. For the ATI 0.19, three peptides P/IS 3, 4, and 22 were available. The same was true for the ATI 0.53, where P/IS 3, 4, and 5 were included in the method. Since P/IS3 and 4 were present in both ATIs 0.19 and 0.53, P/IS22 and P/IS5 were chosen as quantifiers for the ATIs 0.19 and 0.53, respectively. For the other ATIs, two peptides were available each. P/IS1 and 2 were possible quantifiers for the ATI 0.28. The values for both were similar and had a strong correlation (*r* = 0.922), so that P/IS1 was chosen as a quantifier because of the lower limit of detection (LOD) and quantitation (LOQ). Peptides P/IS9 and 10 were available for the quantitation of CM3. The results for both peptides were also strongly correlated (*r* = 0.859), which is why P/IS9 was chosen as a quantifier due to the lower LOD/LOQ and the lower number of amino acids. Quantitation of CM16 was possible with either P/IS11 or P/IS12. Again, the results were highly correlated (*r* = 0.862). P/IS11 was selected as a quantifier because the content of P/IS12 needs to be calculated based on two peptides due to water loss at the N-terminal glutamine after dissolving^24^. For CMX1/2/3, P/IS16 and 21 were available, but since P/IS21 could not be detected in any of the common wheat samples in this study, P/IS16 was used for quantitation of the respective ATIs. P/IS14 and 15 were peptides for WASI, but there was only a weak correlation between the content of these two peptides (*r* = 0.637). P/IS14 was picked as a quantifier because of the smaller number of amino acids and the lower LOD/LOQ. For WCI, P/IS17 and 18 were present. The standard deviations for the samples were lower for P/IS18, although the length of the peptide was longer. Since the contents of both peptides hardly differed (*r* = 0.942) and the LOD and LOQ were lower, P/IS18 was picked as a quantifier. The content of WTI was also strongly correlated for both peptides P/IS19 and 20 (*r* = 0.990). Since both peptides have 10 amino acids, the one with the lower standard deviations and lower LOD and LOQ was picked, P/IS20.

### ATI activity by enzymatic assay

The inhibitory activity against porcine pancreas α-amylase was determined in triplicate according to Jahn et al. ^9^. Extraction buffer (1 mL, 20 mmol/L Na_2_HPO_4_×2 H_2_O, 100 mmol/L NaCl, pH 7.5) was added to 100 mg of flour. After 1 h of magnetic stirring at 22 °C, a centrifugation step was carried out (25 min, 22 °C, 3750 rcf). Then, the supernatant was incubated in a thermal shaker for 20 min (80 °C, 500 rpm) to inactivate endogenous enzymes. The suspension was again centrifuged for 15 min (22 °C, 5000 rcf) and the resulting supernatant was diluted with the extraction buffer (dilution factor 100–200) for the measurement. The DQ™-starch reagent of the assay kit was dissolved in the included sodium acetate buffer (100 μL, 50 mmol/L) and diluted with 900 µL of extraction buffer. Until used, the reagent was stored in the dark and then again diluted 1:10 with the extraction buffer. Porcine pancreas α-amylase was dissolved (1 mg/mL) and diluted (dilution factor 100) in extraction buffer to obtain linear formation of the fluorescent product.

The following solutions were pipetted into the cavities of a 96-well plate: sample (25 µL diluted extract and 25 µL α-amylase solution), blank sample (25 µL diluted extract and 25 µL extraction buffer), positive sample (25 µL extraction buffer and 25 µL α-amylase solution) and blank positive sample (50 µL extraction buffer). After incubation for 10 min, the DQ™-starch reagent (50 µL) was added, and the fluorescence measurement was started immediately. The measurement was carried out using a multiplate reader (Tecan, Maennedorf, Switzerland) at an excitation wavelength of 485 nm and an emission wavelength of 515 nm as a continuous determination over 20 min in intervals of 20 s. The inhibitory activity of each sample was calculated relatively by means of the slopes of the sample and the positive sample, the dilution factor, and the sample weights according to Jahn et al.^9^.

### Statistics

Mean values and standard deviations of triplicates were calculated with Microsoft Office Excel 2016 (Microsoft Corporation, Seattle, WA, USA). One-way and two-way ANOVA with Tukey’s test (*p* ≤ 0.05), PCA and Pearson correlations were performed using OriginPro 2023 (OriginLab, Northampton, MA, USA). The Shapiro-Wilk test confirmed normal distribution for all data. Pearson correlation coefficients (*r*) were defined as follows: ±0.54 < *r* ≤ ±0.67: weak correlation; ±0.67 < *r* ≤ ±0.78: medium correlation; ±0.78 < r ≤ ±1.00: strong correlation^25^.

## Supplementary information

landraces_ati_supplementary material

## Data Availability

All data generated or analyzed during this study are included in this published article and its supplementary information files. Mass spectrometry data are publicly available on Panorama Public (https://panoramaweb.org/7Ml9oX.url).
