# Supplementary material for: Amylase/trypsin-inhibitor content and inhibitory activity of German common wheat landraces and modern varieties do not differ
Source: NPJ Sci Food. 2025 Feb 20;9:24. doi: 10.1038/s41538-025-00385-z (PMC11842761; doi:10.1038/s41538-025-00385-z)
Supplement: Supplementary file 1 — landraces_ati_supplementary material [file 41538_2025_385_MOESM1_ESM.pdf]

Amylase/trypsin-inhibitor content and inhibitory activity of German common wheat landraces and modern varieties do not differ

Nora JAHN[1], Sabrina GEISSLITZ[2], Ulla KONRADL[3], Klaus FLEISSNER[3], Katharina A. SCHERF[1,3,4*]

SUPPLEMENTARY MATERIAL

[1] Department of Bioactive and Functional Food Chemistry, Institute of Applied Biosciences, Karlsruhe Institute of Technology (KIT), Adenauerring 20 a, 76131 Karlsruhe, Germany

[2] Leibniz Institute for Food Systems Biology at the Technical University of Munich, 85354 Freising, Germany

[3] Bavarian State Research Center for Agriculture (LfL), Kleeberg 14, 94099 Ruhstorf a.d.Rott, Germany

[4] Technical University of Munich, TUM School of Life Sciences, Professorship of Food Biopolymer Systems, Freising, Germany

* Corresponding author: k.scherf.leibniz-lsb@tum.de

Nora Jahn: nora.jahn@kit.edu; ORCID: 0000-0003-2626-8585

Sabrina Geisslitz: s.geisslitz.leibniz-lsb@tum.de; ORCID: 0000-0002-6811-4069

Ulla Konradl: ulla.konradl@lfl.bayern.de; ORCID: 0009-0009-4437-0541

Klaus Fleissner: klaus.fleissner@lfl.bayern.de; ORCID: 0009-0001-4779-1028

Katharina Anne Scherf: k.scherf.leibniz-lsb@tum.de; ORCID: 0000-0001-8315-540

Supplementary Table 1. Overview of the common wheat varieties grown in three consecutive years (2021-2023).

Variety	Abbreviation	Group
Ackermanns Bayernkönig	ABK	Landraces
Alpiner begrannter Land	ABL	
Altbanater	ALT	
Berchtesgadener Vogel	BEV	
Schwäbischer Dickkopf Landweizen	SDL	
Roter Sächsischer Landweizen	RSL	
Nördlinger Roter	NOR	
Niederbayerischer Braun	NBR	
Unterfränkischer Land	UNL	
Wahrberger Ruf	WAR	
Wetterauer Fuchs	WEF	
Eglfinger Hohenstaufen	EGH	
Freisinger Landweizen	FLW	
Weihenstephan (Igelweizen)	WEI	
RGT Reform	RGT	Modern varieties
Boss	BOS	
Elixer	ELX	
Wendelin	WEN	
KWS Sharki	KWS	
Wiwa	WIW	

Supplementary Table 2. Amylase/trypsin-inhibitor content of landraces and modern varieties (asterisks) of all three harvest years separately and mean of three years. Values are given in (A) mg/g (B) % of crude protein and (C) % of albumin and globulins. Abbreviations for the varieties can be found in Supplementary Table 1.

A		2021	2022	2023	Mean	B	2021	2022	2023	Mean	C	2021	2022	2023	Mean
	**ABK**	6.6	8.5	5.5	6.8		8.3	8.6	6.8	7.9		46.0	41.0	31.5	39.5
	**ABL**	7.5	8.5	6.8	7.6		8.4	7.3	6.9	7.6		37.2	37.9	32.6	35.9
	**ALT**	6.8	7.7	5.7	6.7		7.8	6.6	6.1	6.8		36.2	36.4	30.1	34.2
	**BEV**	6.4	8.3	6.0	6.9		8.4	6.8	7.6	7.6		36.0	37.0	34.9	36.0
	**SDL**	6.6	9.1	6.3	7.3		6.8	6.7	6.4	6.6		45.5	38.7	31.6	38.6
	**RSL**	5.7	7.6	5.7	6.4		6.9	7.7	7.4	7.4		35.4	39.3	35.1	36.6
	**NOR**	5.8	7.8	5.6	6.4		7.3	6.5	6.8	6.9		36.1	37.2	33.4	35.5
	**NBR**	5.7	8.1	5.4	6.4		7.9	7.5	6.5	7.3		35.7	37.8	31.1	34.9
	**UNL**	5.9	7.3	5.4	6.2		7.0	6.3	6.8	6.7		34.3	35.0	32.5	33.9
	**WAR**	5.9	7.7	6.3	6.6		8.7	7.5	8.4	8.2		36.5	35.5	35.0	35.6
	**WEF**	5.6	9.0	5.6	6.7		7.5	7.6	7.6	7.6		36.4	38.8	34.9	36.7
	**EGH**	7.4	10.3	7.6	8.4		6.3	8.3	6.8	7.1		30.7	41.8	30.5	34.3
	**FLW**	7.0	9.5	6.6	7.7		5.8	7.2	5.6	6.2		31.8	41.0	33.9	35.6
	**WEI**	5.8	7.4	5.1	6.1		4.5	6.8	5.2	5.5		27.2	38.4	29.3	31.6
	**Mean**	6.3	8.3	6.0	6.9		7.3	7.3	6.8	7.1		36.1	38.3	32.6	35.6
	**RGT***	6.6	9.6	7.1	7.8		9.2	9.4	8.8	9.1		32.8	40.9	36.8	36.8
	**BOS***	5.9	9.0	5.7	6.9		8.5	7.4	7.7	7.9		30.7	35.9	34.1	33.6
	**ELX***	5.2	6.8	4.9	5.7		7.0	7.3	7.7	7.3		31.3	34.7	37.6	34.5
	**WEN***	5.5	8.8	6.4	6.9		6.8	6.8	7.2	6.9		29.8	39.0	40.3	36.4
	**KWS***	7.1	10.4	6.7	8.1		6.6	8.1	6.1	7.0		30.0	40.4	33.2	34.5
	**WIW***	n.a.	8.7	6.1	7.4		n.a.	7.0	6.6	6.8		n.a.	38.9	34.5	36.7
	**Mean**	6.1	8.9	6.2	7.1		7.6	7.7	7.4	7.5		30.9	38.3	36.1	35.4

Supplementary Table 3. Crude protein content of landraces and modern varieties (asterisks) of all three harvest years separately and mean of three years. Values are given in %. Data are already published in Jahn et al. 2024. Abbreviations for the varieties can be found in Supplementary Table 1.

	2021	2022	2023	Mean
**ABK**	7.9	9.8	8.1	8.6
**ABL**	8.9	11.5	9.8	10.1
**ALT**	8.7	11.7	9.4	9.9
**BEV**	7.6	12.2	7.8	9.2
**SDL**	9.7	13.6	9.9	11.1
**RSL**	8.3	9.9	7.7	8.6
**NOR**	8.0	11.9	8.2	9.4
**NBR**	7.2	10.8	8.3	8.8
**UNL**	8.3	11.5	8.0	9.3
**WAR**	6.7	10.3	7.5	8.2
**WEF**	7.5	11.8	7.4	8.9
**EGH**	11.8	12.4	11.2	11.8
**FLW**	12.1	13.1	11.7	12.3
**WEI**	13.0	11.0	9.9	11.3
**RGT***	7.2	10.2	8.0	8.5
**BOS***	7.0	12.1	7.4	8.9
**ELX***	7.4	9.3	6.4	7.7
**WEN***	8.2	13.1	8.8	10.0
**KWS***	10.8	12.7	10.9	11.5
**WIW***	n.a.	12.4	9.3	10.8

Supplementary Table 4. Albumin and globulin content of landraces and modern varieties (asterisks) of all three harvest years separately and mean of three years. Values are given in mg/g. Data are already published in Jahn et al. 2024. Abbreviations for the varieties can be found in Supplementary Table 1.

	2021	2022	2023	Mean
ABK	14.3	20.6	17.4	17.4
ABL	20.2	22.3	20.8	21.1
ALT	18.8	21.2	19.0	19.6
BEV	17.7	22.3	17.1	19.1
SDL	14.5	23.4	20.1	19.3
RSL	16.2	19.4	16.3	17.3
NOR	16.1	20.9	16.8	18.0
NBR	15.9	21.4	17.3	18.2
UNL	17.1	20.8	16.7	18.2
WAR	16.1	21.8	18.1	18.7
WEF	15.5	23.1	16.2	18.3
EGH	24.2	24.7	24.8	24.6
FLW	22.0	23.1	19.3	21.5
WEI	21.5	19.3	17.5	19.4
RGT*	20.2	23.4	19.2	21.0
BOS*	19.3	24.9	16.9	20.4
ELX*	16.6	19.7	13.1	16.5
WEN*	18.6	22.6	15.8	19.0
KWS*	23.8	25.6	20.0	23.2
WIW*	n.a.	22.3	17.8	20.0

Supplementary Table 5. Content of 0.19 of landraces and modern varieties (asterisks) of all three harvest years separately and mean of three years. Values are given in (A) µg/g (B) % of total amylase/trypsin-inhibitor content. Abbreviations for the varieties can be found in Supplementary Table 1.

A		2021	2022	2023	Mean	B	2021	2022	2023	Mean
	**ABK**	1872	2501	1545	1973		28.6	29.6	28.2	28.8
	**ABL**	1955	2327	1732	2004		26.0	27.5	25.5	26.3
	**ALT**	2118	2408	1820	2115		31.2	31.3	31.9	31.5
	**BEV**	1956	2800	1863	2206		30.7	33.9	31.2	31.9
	**SDL**	2175	2902	2010	2362		33.0	32.0	31.7	32.2
	**RSL**	1790	2382	1616	1929		31.2	31.3	28.2	30.2
	**NOR**	1809	2575	1672	2019		31.1	33.2	29.8	31.4
	**NBR**	1701	2619	1677	1999		29.9	32.4	31.2	31.2
	**UNL**	1774	2321	1462	1852		30.2	31.9	26.9	29.7
	**WAR**	1567	2331	1706	1868		26.7	30.2	27.0	28.0
	**WEF**	1761	2945	1623	2109		31.3	32.9	28.8	31.0
	**EGH**	2449	3373	2595	2806		33.0	32.7	34.2	33.3
	**FLW**	2410	2913	2291	2538		34.4	30.8	35.0	33.4
	**WEI**	2281	2777	1968	2342		39.1	37.4	38.5	38.3
	**Mean**	1973	2655	1827	2152		31.2	31.9	30.6	31.2
	**RGT***	1702	2902	1996	2200		25.7	30.3	28.2	28.1
	**BOS***	1755	2770	1602	2042		29.6	31.0	27.9	29.5
	**ELX***	1880	2528	1698	2035		36.2	37.1	34.5	35.9
	**WEN***	1702	3164	2095	2320		30.8	35.9	32.9	33.2
	**KWS***	2168	3329	2286	2594		30.4	32.2	34.4	32.3
	**WIW***	n.a.	2798	1858	2328		n.a.	32.3	30.3	31.3
	**Mean**	1842	2915	1923	2253		30.5	33.1	31.4	31.7

Supplementary Table 6. Content of CM3 of landraces and modern varieties (asterisks) of all three harvest years separately and mean of three years. Values are given in (A) µg/g (B) % of total amylase/trypsin-inhibitor content. Abbreviations for the varieties can be found in Supplementary Table 1.

A		2021	2022	2023	Mean	B	2021	2022	2023	Mean
	**ABK**	1032	1172	811	1005		15.8	13.9	14.8	14.8
	**ABL**	1131	1158	921	1070		15.0	13.7	13.6	14.1
	**ALT**	1004	1067	780	950		14.8	13.9	13.7	14.1
	**BEV**	1005	1047	857	970		15.8	12.7	14.3	14.3
	**SDL**	948	1174	936	1019		14.4	12.9	14.8	14.0
	**RSL**	849	1025	813	896		14.8	13.5	14.2	14.1
	**NOR**	892	1017	835	915		15.3	13.1	14.9	14.4
	**NBR**	929	1118	797	948		16.4	13.8	14.8	15.0
	**UNL**	890	906	810	869		15.1	12.5	14.9	14.2
	**WAR**	944	1035	1020	1000		16.1	13.4	16.2	15.2
	**WEF**	824	1329	812	988		14.6	14.8	14.4	14.6
	**EGH**	1074	1447	1043	1188		14.5	14.0	13.8	14.1
	**FLW**	945	1373	899	1072		13.5	14.5	13.7	13.9
	**WEI**	675	851	504	676		11.6	11.5	9.8	11.0
	**Mean**	939	1123	846	969		14.8	13.4	14.1	14.1
	**RGT***	1127	1396	1166	1230		17.0	14.6	16.5	16.0
	**BOS***	876	1247	864	996		14.8	13.9	15.0	14.6
	**ELX***	678	853	698	743		13.0	12.5	14.2	13.2
	**WEN***	758	1077	893	909		13.7	12.2	14.0	13.3
	**KWS***	1046	1485	925	1152		14.7	14.4	13.9	14.3
	**WIW***	n.a.	1258	934	1096		n.a.	14.5	15.2	14.9
	**Mean**	897	1219	913	1021		14.6	13.7	14.8	14.4

Supplementary Table 7. Content of CM17 of landraces and modern varieties (asterisks) of all three harvest years separately and mean over three years. Values are given in (A) µg/g (B) % of total amylase/trypsin-inhibitor content. Abbreviations for the varieties can be found in Supplementary Table 1.

A		2021	2022	2023	Mean	B	2021	2022	2023	Mean
	**ABK**	619	986	544	716		9.5	11.7	9.9	10.3
	**ABL**	785	1210	797	930		10.4	14.3	11.7	12.2
	**ALT**	665	1024	571	753		9.8	13.3	10.0	11.0
	**BEV**	594	966	589	716		9.3	11.7	9.9	10.3
	**SDL**	643	1156	628	809		9.8	12.7	9.9	10.8
	**RSL**	574	956	606	712		10.0	12.5	10.6	11.0
	**NOR**	568	1004	610	727		9.8	12.9	10.9	11.2
	**NBR**	571	1090	527	730		10.1	13.5	9.8	11.1
	**UNL**	565	898	597	687		9.6	12.4	11.0	11.0
	**WAR**	579	873	590	681		9.9	11.3	9.3	10.2
	**WEF**	556	1067	572	732		9.9	11.9	10.2	10.6
	**EGH**	765	1303	727	932		10.3	12.6	9.6	10.8
	**FLW**	652	1230	582	821		9.3	13.0	8.9	10.4
	**WEI**	461	827	414	567		7.9	11.1	8.1	9.0
	**Mean**	614	1042	597	751		9.7	12.5	10.0	10.7
	**RGT***	706	1254	745	902		10.6	13.1	10.5	11.4
	**BOS***	536	1068	544	716		9.1	11.9	9.5	10.2
	**ELX***	470	760	422	551		9.0	11.2	8.6	9.6
	**WEN***	475	998	606	693		8.6	11.3	9.5	9.8
	**KWS***	758	1349	591	899		10.6	13.0	8.9	10.9
	**WIW***	n.a.	1079	594	836		n.a.	12.5	9.7	11.1
	**Mean**	589	1085	584	766		9.6	12.2	9.5	10.5

Supplementary Table 8. Content of CM16 of landraces and modern varieties (asterisks) of all three harvest years separately and mean of three years. Values are given in (A) µg/g (B) % of total amylase/trypsin-inhibitor content. Abbreviations for the varieties can be found in Supplementary Table 1.

A		2021	2022	2023	Mean	B	2021	2022	2023	Mean
	**ABK**	688	778	586	684		10.5	9.2	10.7	10.1
	**ABL**	946	897	839	894		12.6	10.6	12.4	11.9
	**ALT**	785	741	628	718		11.6	9.6	11.0	10.7
	**BEV**	595	726	666	663		9.3	8.8	11.2	9.8
	**SDL**	659	859	695	738		10.0	9.5	11.0	10.1
	**RSL**	519	662	570	584		9.1	8.7	9.9	9.2
	**NOR**	581	661	607	617		10.0	8.5	10.8	9.8
	**NBR**	570	690	551	604		10.0	8.5	10.3	9.6
	**UNL**	636	628	554	606		10.8	8.6	10.2	9.9
	**WAR**	562	663	591	605		9.6	8.6	9.4	9.2
	**WEF**	519	926	521	655		9.2	10.3	9.2	9.6
	**EGH**	705	823	688	739		9.5	8.0	9.1	8.9
	**FLW**	702	887	662	750		10.0	9.4	10.1	9.8
	**WEI**	526	617	441	528		9.0	8.3	8.6	8.6
	**Mean**	642	754	614	670		10.1	9.1	10.3	9.8
	**RGT***	640	848	725	738		9.7	8.9	10.3	9.6
	**BOS***	601	823	588	671		10.2	9.2	10.2	9.9
	**ELX***	536	552	470	520		10.3	8.1	9.6	9.3
	**WEN***	553	665	605	608		10.0	7.5	9.5	9.0
	**KWS***	690	900	589	727		9.7	8.7	8.9	9.1
	**WIW***	n.a.	773	614	694		n.a.	8.9	10.0	9.5
	**Mean**	604	760	599	651		10.0	8.6	9.7	9.4

Supplementary Table 9. Content of 0.28 of landraces and modern varieties (asterisks) of all three harvest years separately and mean of three years. Values are given in (A) µg/g (B) % of total amylase/trypsin-inhibitor content. Abbreviations for the varieties can be found in Supplementary Table 1.

A		2021	2022	2023	Mean	B	2021	2022	2023	Mean
	**ABK**	681	906	604	730		10.4	10.7	11.0	10.7
	**ABL**	764	842	708	771		10.1	10.0	10.4	10.2
	**ALT**	625	673	653	651		9.2	8.7	11.5	9.8
	**BEV**	649	776	666	697		10.2	9.4	11.2	10.2
	**SDL**	625	812	701	713		9.5	8.9	11.1	9.8
	**RSL**	640	804	750	731		11.2	10.6	13.1	11.6
	**NOR**	549	635	599	594		9.4	8.2	10.7	9.4
	**NBR**	581	671	513	588		10.2	8.3	9.5	9.4
	**UNL**	576	744	607	642		9.8	10.2	11.2	10.4
	**WAR**	637	877	739	751		10.9	11.3	11.7	11.3
	**WEF**	665	545	726	646		11.8	6.1	12.9	10.3
	**EGH**	721	907	826	818		9.7	8.8	10.9	9.8
	**FLW**	717	814	702	744		10.2	8.6	10.7	9.9
	**WEI**	589	687	619	632		10.1	9.3	12.1	10.5
	**Mean**	644	764	672	693		10.2	9.2	11.3	10.2
	**RGT***	532	687	507	575		8.0	7.2	7.2	7.5
	**BOS***	497	671	592	587		8.4	7.5	10.3	8.7
	**ELX***	430	529	426	462		8.3	7.8	8.7	8.2
	**WEN***	668	872	765	768		12.1	9.9	12.0	11.3
	**KWS***	671	842	652	722		9.4	8.1	9.8	9.1
	**WIW***	n.a.	732	664	698		n.a.	8.5	10.8	9.6
	**Mean**	560	722	601	635		9.2	8.2	9.8	9.1

Supplementary Table 10. Content of CM2 of landraces and modern varieties (asterisks) of all three harvest years separately and mean of three years. Values are given in (A) µg/g (B) % of total amylase/trypsin-inhibitor content. Abbreviations for the varieties can be found in Supplementary Table 1.

A		2021	2022	2023	Mean	B	2021	2022	2023	Mean
	**ABK**	471	702	436	536		7.2	8.3	8.0	7.8
	**ABL**	594	723	596	637		7.9	8.6	8.8	8.4
	**ALT**	518	678	400	532		7.6	8.8	7.0	7.8
	**BEV**	492	694	456	547		7.7	8.4	7.6	7.9
	**SDL**	496	798	472	589		7.5	8.8	7.4	7.9
	**RSL**	348	570	398	438		6.1	7.5	6.9	6.8
	**NOR**	420	653	391	488		7.2	8.4	7.0	7.5
	**NBR**	420	707	405	511		7.4	8.7	7.5	7.9
	**UNL**	454	586	474	505		7.7	8.1	8.7	8.2
	**WAR**	436	698	528	554		7.4	9.0	8.4	8.3
	**WEF**	359	928	388	558		6.4	10.4	6.9	7.9
	**EGH**	575	912	508	665		7.7	8.8	6.7	7.8
	**FLW**	543	832	465	614		7.8	8.8	7.1	7.9
	**WEI**	408	590	310	436		7.0	8.0	6.1	7.0
	**Mean**	467	719	445	544		7.3	8.6	7.4	7.8
	**RGT***	580	863	617	687		8.7	9.0	8.7	8.8
	**BOS***	454	820	437	570		7.7	9.2	7.6	8.2
	**ELX***	354	545	336	412		6.8	8.0	6.8	7.2
	**WEN***	397	698	457	517		7.2	7.9	7.2	7.4
	**KWS***	540	897	460	632		7.6	8.7	6.9	7.7
	**WIW***	n.a.	n.a.	466	611		n.a.	n.a.	7.6	8.2
	**Mean**	465	763	462	572		7.6	8.6	7.5	7.9

Supplementary Table 11. Content of CM1 of landraces and modern varieties (asterisks) of all three harvest years separately and mean of three years. Values are given in (A) µg/g (B) % of total amylase/trypsin-inhibitor content. Abbreviations for the varieties can be found in Supplementary Table 1.

A		2021	2022	2023	Mean	B	2021	2022	2023	Mean
	**ABK**	345	446	274	355		5.3	5.3	5.0	5.2
	**ABL**	444	472	396	437		5.9	5.6	5.8	5.8
	**ALT**	343	389	283	338		5.1	5.1	5.0	5.0
	**BEV**	295	401	293	330		4.6	4.9	4.9	4.8
	**SDL**	317	482	339	379		4.8	5.3	5.3	5.2
	**RSL**	249	357	264	290		4.4	4.7	4.6	4.5
	**NOR**	304	401	286	330		5.2	5.2	5.1	5.2
	**NBR**	304	422	315	347		5.4	5.2	5.9	5.5
	**UNL**	283	350	275	303		4.8	4.8	5.1	4.9
	**WAR**	288	372	351	337		4.9	4.8	5.6	5.1
	**WEF**	246	437	254	312		4.4	4.9	4.5	4.6
	**EGH**	352	480	333	388		4.7	4.7	4.4	4.6
	**FLW**	398	455	299	384		5.7	4.8	4.6	5.0
	**WEI**	234	326	204	255		4.0	4.4	4.0	4.1
	**Mean**	314	414	297	342		4.9	5.0	5.0	5.0
	**RGT***	399	477	412	429		6.0	5.0	5.8	5.6
	**BOS***	308	403	298	336		5.2	4.5	5.2	5.0
	**ELX***	288	350	296	311		5.5	5.1	6.0	5.6
	**WEN***	294	415	319	342		5.3	4.7	5.0	5.0
	**KWS***	396	552	308	419		5.6	5.3	4.6	5.2
	**WIW***	n.a.	399	310	354		n.a.	4.6	5.1	4.8
	**Mean**	337	432	324	365		5.5	4.9	5.3	5.2

Supplementary Table 12. Content of 0.53 of landraces and modern varieties (asterisks) of all three harvest years separately and mean of three years. Values are given in (A) µg/g (B) % of total amylase/trypsin-inhibitor content. Abbreviations for the varieties can be found in Supplementary Table 1.

A		2021	2022	2023	Mean	B	2021	2022	2023	Mean
	**ABK**	312	441	273	342		4.8	5.2	5.0	5.0
	**ABL**	364	322	304	330		4.8	3.8	4.5	4.4
	**ALT**	316	315	211	281		4.7	4.1	3.7	4.2
	**BEV**	265	432	201	300		4.2	5.2	3.4	4.3
	**SDL**	251	455	189	298		3.8	5.0	3.0	3.9
	**RSL**	295	399	277	324		5.2	5.2	4.8	5.1
	**NOR**	341	450	293	362		5.9	5.8	5.2	5.6
	**NBR**	239	389	277	302		4.2	4.8	5.1	4.7
	**UNL**	256	357	202	272		4.4	4.9	3.7	4.3
	**WAR**	349	377	253	326		5.9	4.9	4.0	4.9
	**WEF**	261	413	272	316		4.6	4.6	4.8	4.7
	**EGH**	354	484	326	388		4.8	4.7	4.3	4.6
	**FLW**	360	441	280	360		5.1	4.7	4.3	4.7
	**WEI**	401	368	387	385		6.9	5.0	7.6	6.5
	**Mean**	312	403	268	328		4.9	4.9	4.5	4.8
	**RGT***	271	452	271	331		4.1	4.7	3.8	4.2
	**BOS***	316	482	235	344		5.3	5.4	4.1	4.9
	**ELX***	268	264	187	240		5.2	3.9	3.8	4.3
	**WEN***	347	485	211	348		6.3	5.5	3.3	5.0
	**KWS***	384	409	369	387		5.4	4.0	5.5	5.0
	**WIW***	n.a.	365	237	301		n.a.	4.2	3.9	4.0
	**Mean**	317	409	252	325		5.3	4.6	4.1	4.6

Supplementary Table 13. Content of CMX1/2/3 of landraces and modern varieties (asterisks) of all three harvest years separately and mean of three years. Values are given in (A) µg/g (B) % of total amylase/trypsin-inhibitor content. Abbreviations for the varieties can be found in Supplementary Table 1.

A		2021	2022	2023	Mean	B	2021	2022	2023	Mean
	**ABK**	228	223	182	211		3.5	2.6	3.3	3.2
	**ABL**	230	220	195	215		3.1	2.6	2.9	2.9
	**ALT**	162	149	133	148		2.4	1.9	2.3	2.2
	**BEV**	226	161	144	177		3.5	2.0	2.4	2.6
	**SDL**	145	167	121	144		2.2	1.8	1.9	2.0
	**RSL**	216	253	241	237		3.8	3.3	4.2	3.8
	**NOR**	191	200	170	187		3.3	2.6	3.0	3.0
	**NBR**	173	206	172	184		3.1	2.6	3.2	2.9
	**UNL**	244	227	220	230		4.2	3.1	4.1	3.8
	**WAR**	228	221	243	231		3.9	2.9	3.8	3.5
	**WEF**	201	187	265	218		3.6	2.1	4.7	3.5
	**EGH**	243	291	273	269		3.3	2.8	3.6	3.2
	**FLW**	129	232	164	175		1.8	2.5	2.5	2.3
	**WEI**	131	180	110	140		2.3	2.4	2.1	2.3
	**Mean**	196	208	188	198		3.1	2.5	3.2	2.9
	**RGT***	125	149	118	130		1.9	1.6	1.7	1.7
	**BOS***	196	233	196	208		3.3	2.6	3.4	3.1
	**ELX***	136	158	143	145		2.6	2.3	2.9	2.6
	**WEN***	126	166	155	149		2.3	1.9	2.4	2.2
	**KWS***	230	297	230	252		3.2	2.9	3.5	3.2
	**WIW***	n.a.	250	229	240		n.a.	2.9	3.7	3.3
	**Mean**	162	209	178	188		2.7	2.4	2.9	2.7

Supplementary Table 14. Content of WCI of landraces and modern varieties (asterisks) of all three harvest years separately and mean of three years. Values are given in (A) µg/g (B) % of total amylase/trypsin-inhibitor content. Abbreviations for the varieties can be found in Supplementary Table 1.

A		2021	2022	2023	Mean	B	2021	2022	2023	Mean
	**ABK**	215	217	182	204		3.3	2.6	3.3	3.1
	**ABL**	259	205	226	230		3.4	2.4	3.3	3.1
	**ALT**	196	181	162	180		2.9	2.4	2.9	2.7
	**BEV**	200	168	177	182		3.1	2.0	3.0	2.7
	**SDL**	194	185	191	190		2.9	2.0	3.0	2.7
	**RSL**	139	158	157	151		2.4	2.1	2.7	2.4
	**NOR**	111	110	106	109		1.9	1.4	1.9	1.7
	**NBR**	116	123	102	114		2.0	1.5	1.9	1.8
	**UNL**	161	156	162	160		2.7	2.2	3.0	2.6
	**WAR**	171	184	204	186		2.9	2.4	3.2	2.8
	**WEF**	141	132	157	143		2.5	1.5	2.8	2.3
	**EGH**	165	219	186	190		2.2	2.1	2.5	2.3
	**FLW**	133	204	148	162		1.9	2.2	2.3	2.1
	**WEI**	95	151	107	118		1.6	2.0	2.1	1.9
	**Mean**	164	171	162	166		2.6	2.1	2.7	2.4
	**RGT***	251	260	250	254		3.8	2.7	3.5	3.3
	**BOS***	219	221	195	212		3.7	2.5	3.4	3.2
	**ELX***	136	180	150	155		2.6	2.6	3.0	2.8
	**WEN***	188	197	184	190		3.4	2.2	2.9	2.8
	**KWS***	178	224	180	194		2.5	2.2	2.7	2.5
	**WIW***	n.a.	200	173	186		n.a.	2.3	2.8	2.6
	**Mean**	194	214	189	198		3.2	2.4	3.1	2.9

Supplementary Table 15. Content of WASI of landraces and modern varieties (asterisks) of all three harvest years separately and mean of three years. Values are given in (A) µg/g (B) % of total amylase/trypsin-inhibitor content. Abbreviations for the varieties can be found in Supplementary Table 1.

A		2021	2022	2023	Mean	B	2021	2022	2023	Mean
	**ABK**	41	48	33	41		0.6	0.6	0.6	0.6
	**ABL**	38	46	43	42		0.5	0.6	0.6	0.6
	**ALT**	36	50	39	42		0.5	0.7	0.7	0.6
	**BEV**	37	56	43	46		0.6	0.7	0.7	0.7
	**SDL**	37	69	48	51		0.6	0.8	0.8	0.7
	**RSL**	28	43	38	36		0.5	0.6	0.7	0.6
	**NOR**	30	46	32	36		0.5	0.6	0.6	0.6
	**NBR**	30	40	34	34		0.5	0.5	0.6	0.6
	**UNL**	36	55	41	44		0.6	0.8	0.8	0.7
	**WAR**	39	63	51	51		0.7	0.8	0.8	0.8
	**WEF**	28	39	37	34		0.5	0.4	0.7	0.5
	**EGH**	44	52	66	54		0.6	0.5	0.9	0.7
	**FLW**	51	40	43	45		0.7	0.4	0.7	0.6
	**WEI**	40	43	51	44		0.7	0.6	1.0	0.8
	**Mean**	37	49	43	43		0.6	0.6	0.7	0.6
	**RGT***	34	46	47	42		0.5	0.5	0.7	0.6
	**BOS***	43	45	42	43		0.7	0.5	0.7	0.7
	**ELX***	47	51	52	50		0.9	0.8	1.1	0.9
	**WEN***	47	47	44	46		0.8	0.5	0.7	0.7
	**KWS***	41	47	57	48		0.6	0.5	0.9	0.6
	**WIW***	n.a.	38	44	41		n.a.	0.4	0.7	0.6
	**Mean**	42	46	48	45		0.7	0.5	0.8	0.7

Supplementary Table 16. Content of WTI of landraces and modern varieties (asterisks) of all three harvest years separately and mean of three years. Values are given in (A) µg/g (B) % of total amylase/trypsin-inhibitor content. Abbreviations for the varieties can be found in Supplementary Table 1.

A		2021	2022	2023	Mean	B	2021	2022	2023	Mean
	**ABK**	36	52	34	41		0.5	0.6	0.6	0.6
	**ABL**	59	56	53	56		0.8	0.7	0.8	0.8
	**ALT**	56	48	40	48		0.8	0.6	0.7	0.7
	**BEV**	60	38	37	45		0.9	0.5	0.6	0.7
	**SDL**	20	37	24	27		0.3	0.4	0.4	0.4
	**RSL**	19	22	20	20		0.3	0.3	0.4	0.3
	**NOR**	15	13	15	14		0.3	0.2	0.3	0.2
	**NBR**	12	15	15	14		0.2	0.2	0.3	0.2
	**UNL**	55	69	69	64		0.9	0.9	1.3	1.1
	**WAR**	57	69	74	67		1.0	0.9	1.2	1.0
	**WEF**	14	19	23	19		0.2	0.2	0.4	0.3
	**EGH**	18	39	16	24		0.3	0.4	0.2	0.3
	**FLW**	25	59	25	36		0.4	0.6	0.4	0.5
	**WEI**	8	16	8	11		0.1	0.2	0.2	0.2
	**Mean**	32	39	32	35		0.5	0.5	0.5	0.5
	**RGT***	350	448	404	401		5.3	4.7	5.7	5.2
	**BOS***	257	302	276	278		4.3	3.4	4.8	4.2
	**ELX***	61	88	81	77		1.2	1.3	1.7	1.4
	**WEN***	64	78	60	67		1.2	0.9	0.9	1.0
	**KWS***	15	32	13	20		0.2	0.3	0.2	0.2
	**WIW***	n.a.	30	26	28		n.a.	0.4	0.4	0.4
	**Mean**	149	163	143	145		2.4	1.8	2.3	2.1

Supplementary Table 17. Inhibitory activity of landraces and modern varieties (asterisks) of all three harvest years separately and mean of three years. Values are given in AIU/g. Abbreviations for the varieties can be found in Supplementary Table 1.

	2021	2022	2023	Mean
**ABK**	606	534	459	533
**ABL**	639	647	616	634
**ALT**	591	542	507	546
**BEV**	805	668	482	652
**SDL**	912	553	711	725
**RSL**	559	575	653	595
**NOR**	659	526	633	606
**NBR**	764	607	674	682
**UNL**	661	605	704	657
**WAR**	635	681	809	708
**WEF**	554	606	632	597
**EGH**	635	662	720	672
**FLW**	690	566	720	659
**WEI**	610	528	472	537
**Mean**	666	593	628	629
**RGT***	666	647	708	674
**BOS***	662	828	776	755
**ELX***	469	463	482	471
**WEN***	542	562	602	569
**KWS***	766	668	778	737
**WIW***	n.a.	625	709	667
**Mean**	621	632	676	646

Supplementary Table 18. Analysis of variance (ANOVA) table for two-way ANOVA with the factors harvest year and variety for (A) ATI content and (B) inhibitory activity.

	DF	Sum of Squares	Mean Square	F Value	P Value
**A**					
**Harvest year**	2	66.6	33.3	52.7048	<0.0001
**Variety**	1	0.3	0.3	0.4530	0.5038
**Interaction**	2	1.2	0.6	0.9662	0.3871
**Model**	5	74.5	14.9	23.5935	<0.0001
**Error**	53	33.5	0.6		
**Corrected Total**	58	108.0			
**B**					
**Harvest year**	2	14376.6	7188.3	0.7398	0.4821
**Variety**	1	2408.8	2408.8	0.2479	0.6206
**Interaction**	2	20101.7	10050.8	1.0344	0.3625
**Model**	5	49684.7	9936.9	1.0226	0.4138
**Error**	53	514999.3	9717.0		
**Corrected Total**	58	564684.0			

Supplementary Table 19. Pearson correlation analysis of Amylase/trypsin-inhibitor (ATI) content and inhibitory activity of (A) all samples of all three years and (B) the ATIs that mainly inhibit α-amylase (all ATIs except wheat amylase subtilisin inhibitor, wheat trypsin inhibitor, wheat chymotrypsin inhibitor and CMX1/2/3). CM: chloroform/methanol.

	N	Mean	SD	Sum	Min	Max	Pearson correlation coefficient	p-value
**A**								
**ATI content**	59	6.9	1.4	409.1	4.9	10.3	0.07423	0.57632
**Inhibitory activity**	59	633.3	98.7	37362.7	459.0	911.7		
**B**								
**ATI content**	20	5800.0	574.8	115999.7	4767.9	7068.3	0.50324	0.02371
**Inhibitory activity**	20	633.8	75.6	12676.5	471.4	755.0		

Supplementary Table 20. Limit of detection (LOD) and limit of quantitation (LOQ) of the different Amylase/trypsin-inhibitors (ATIs) in µg/g. CM: chloroform/methanol; WASI: wheat amylase subtilisin inhibitor; WCI: wheat chymotrypsin inhibitor; WTI: wheat trypsin inhibitor. *P, proline (13C5, 15N); *V, valine (13C5, 15N); *K, lysine (13C6, 15N2); *R, arginine (13C6, 15N4)

Peptide (P)	ATI	Amino acid sequence	LOD	LOQ
**P1**	0.28	LQCVGSQV*PEA*VLR	0.61	2.02
**P2**	0.28	LTAASVPEVC*K	6.35	21.16
**P3**	0.19 + 0.53	LQCNGSQV*PEA*VLR	13.29	44.30
**P4**	0.19 + 0.53	LTAASITAVC*R	6.87	22.90
**P5**	0.53	EHGVSEGQAGTGAFPSC*R	13.94	46.47
**P6**	CM1	SDPNSSVL*K	0.55	1.84
**P7**	CM2	EYVAQQTCGVGIVGSPVSTE*P*GNT *PR	n.a.	n.a.
**P8**	CM2	TSDPNSGVL*K	2.55	8.51
**P9**	CM3	YFIALPVPSQPVDP*R	1.84	6.14
**P10**	CM3	SGNVGESGLIDL*PGC*PR	3.34	11.14
**P11**	CM16	DYVEQQAC*R	6.98	23.27
**P12**	CM16	QQCCGELANIPQQC*R	3.52	11.73
**P12c**	CM16	QQCCGELANIPQQC*R	3.45	11.50
**P13**	CM17	NYVEEQAC*R	1.97	6.58
**P14**	WASI	HVITGPV*R	2.54	8.47
**P15**	WASI	YSGAEVHEY*K	50.96	169.86
**P16**	CMX1/2/3	EFIAGIVG*R	0.57	1.90
**P17**	WCI	ELAAISSNC*R	6.28	20.92
**P18**	WCI	AFPPSQSQGGGPPQPPLAP*R	1.56	5.19
**P19**	WTI	ELEAVSEEC*R	6.01	20.04
**P20**	WTI	LEGVPEGCT*R	1.80	5.99
**P21**	CMX1/2/3	GSLLQDMS*R	1.67	5.56
**P22**	0.19	EHGAQEGQAGTGAFP*R	16.53	55.11

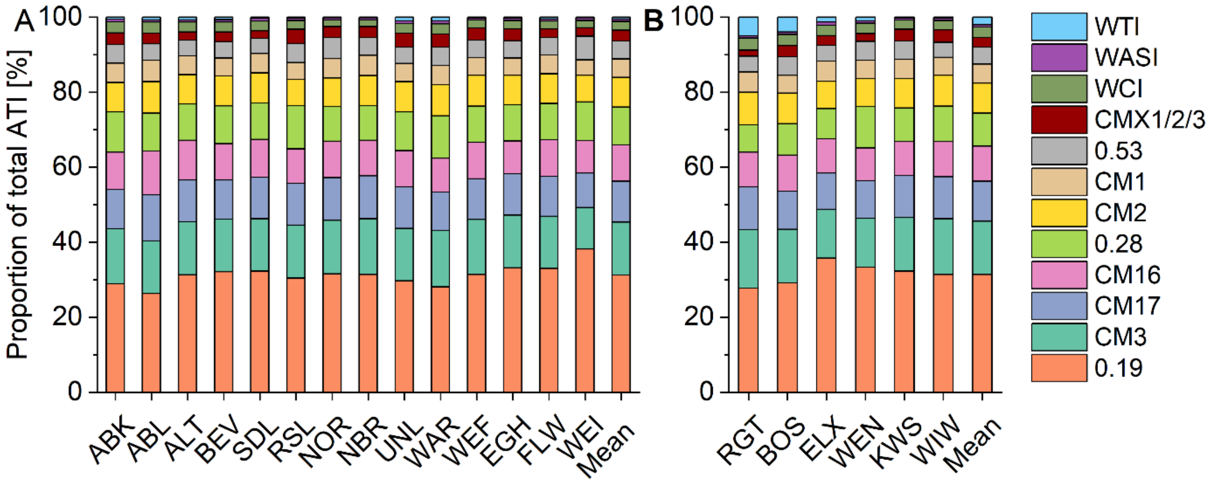

Supplementary Figure 1. Amylase/trypsin-inhibitor (ATI) proportions of single ATIs of (A) landraces and (B) modern varieties. Abbreviations for the varieties can be found in Supplementary Table 1. CM: chloroform/methanol; WASI: wheat amylase subtilisin inhibitor; WCI: wheat chymotrypsin inhibitor; WTI: wheat trypsin inhibitor.

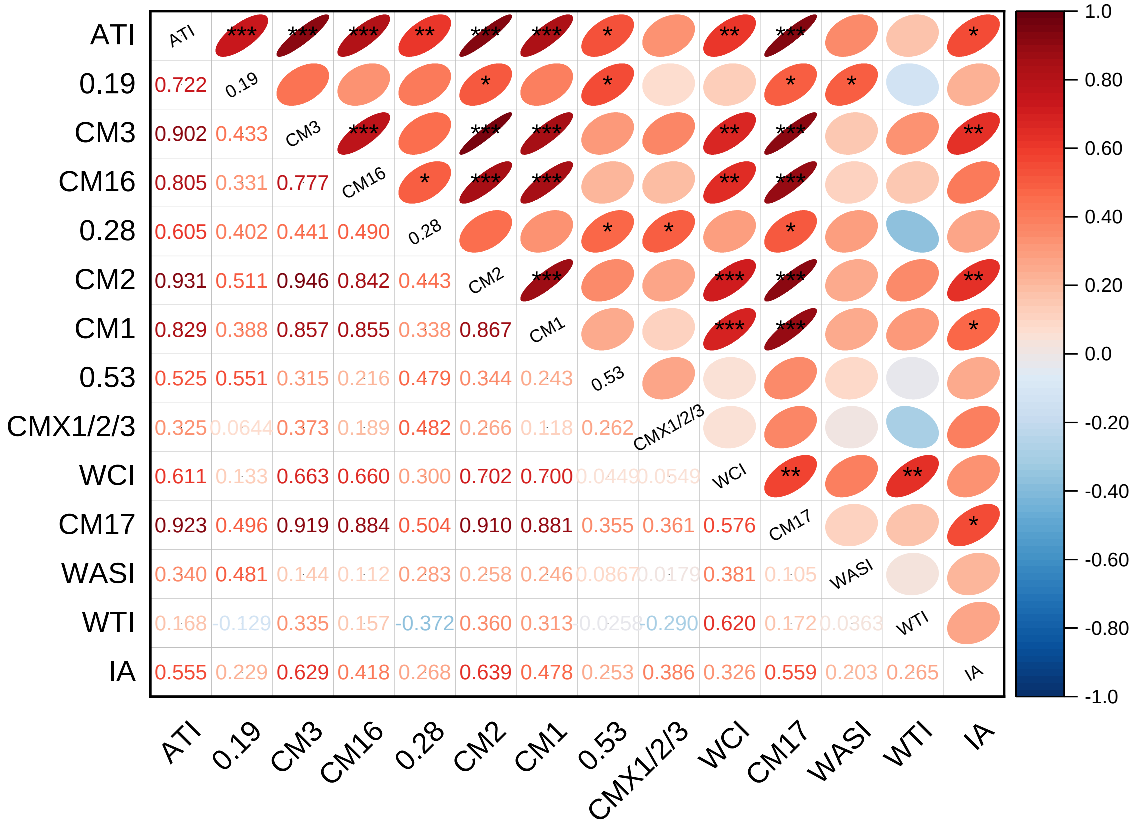

* p<=0.05 ** p<=0.01 *** p<=0.001

Supplementary Figure 2. Correlation matrix of all analyzed parameters of the samples (mean values of three years). Amylase/trypsin-inhibitor content (ATI), the content of all single ATIs and the inhibitory activity (IA) against porcine pancreas α-amylase. CM: chloroform/methanol; WASI: wheat amylase subtilisin inhibitor; WCI: wheat chymotrypsin inhibitor; WTI: wheat trypsin inhibitor.
